# Relationship between Retinal Vascular Caliber and Coronary Artery Disease in Patients with Non-Alcoholic Fatty Liver Disease (NAFLD)

**DOI:** 10.3390/ijerph10083409

**Published:** 2013-08-06

**Authors:** Pikkel Josef, Ibrahim Ali, Prober Ariel, Marmor Alon, Assy Nimer

**Affiliations:** 1 Department of Ophtalmology, Ziv Medical Centre, Safed 13100, Israel; E-Mails: pikel.y@ziv.health.gov.il (P.J.); ibrahim.a@ziv.health.gov.il (I.A.); 2 Faculty of Medicine at Galilee, Bar Ilan University, Safed 13100, Israel; E-Mail: marmor.a@ziv.health.gov.il; 3 Liver Unit, Ziv Medical Centre, Safed 13100, Israel; 4 Department of Radiology, Safed 13100, Israel; E-Mail: Prober.a@ziv.health.gov.il

**Keywords:** cardiovascular disease, fatty liver, retinal vascular calibre, retinal artery, retinal vein, cardiac CT, intimae media thickening

## Abstract

*Objective*: To evaluate the relationship between retinal vascular caliber and cardiovascular disease in non-alcoholic fatty liver disease (NAFLD) patients without diabetes and hypertension. *Methods*: Intention to treat study of individuals who underwent cardiac computed tomography (CT) during a two year period. Coronary artery disease (CAD) was defined as stenosis of >50% in at least one major coronary artery. Liver and spleen density were measured by abdominal (CT); intima-media thickness (IMT) by Doppler ultrasound; retinal artery and vein diameter by colored-retinal angiography; and metabolic syndrome by ATP III guidelines. Serum biomarkers of insulin resistance, inflammation, and oxidant-antioxidant status were assessed. *Results*: Compared with 22 gender and age matched controls, the 29 NAFLD patients showed higher prevalence of coronary plaques (70% *vs.* 30%, *p* < 0.001), higher prevalence of coronary stenosis (30% *vs.* 15%, *p* < 0.001), lower retinal arteriole-to-venule ratio (AVR) (0.66 ± 0.06 *vs.* 0.71 ± 0.02, *p* < 0.01), higher IMT (0.98 ± 0.3 *vs.* 0.83 ± 0.1, *p* < 0.04), higher carotid plaques (60% *vs.* 40%, *p* < 0.001), higher homeostasis model assessment of insulin resistance (HOMA) (4.0 ± 3.4 *vs.* 2.0 ± 1.0, *p* < 0.005), and higher triglyceride levels (200 ± 80 *vs.* 150 ± 60, *p* < 0.005) than controls. Multivariate analysis showed fatty liver (OR 2.5; *p* < 0.01), IMT (OR 2.3 *p* < 0.001), and retinal AVR ratio (OR 1.5, *p* < 0.01) to be strongly associated with CAD independent of metabolic syndrome (OR 1.2, *p* < 0.05). *Conclusions*: Patients with smaller retinal AVR (<0.7) are likely to be at increased risk for CAD and carotid atherosclerosis in patients with NAFLD even without hypertension or diabetes.

## 1. Introduction

Nonalcoholic fatty liver disease (NAFLD) is increasingly recognized as the most common liver disorder in Western countries. It is the most common cause of liver enzyme abnormalities in clinical practice, with a prevalence of 46% in the general population, increasing steadily to 70%–90% in obese and type 2 diabetic patients [1,2]. The pathogenesis of NAFLD includes four major mechanisms: lipotoxicity of fatty acids, insulin resistance, systemic inflammation, and increased oxidative stress and lipid peroxidation [3,4]. Recently, elevated prothrombotic factors that aggravate atherosclerosis have been implicated in nonalcoholic steatohepatitis (NASH) [5,6], the evolutive counterpart of NAFLD. Liver biopsy, the gold standard for diagnosing NASH, is performed sparingly in clinical practice. CT scanning has no role in clinical practice, and ultrasound (US) represents the first line imaging technique and may be particularly useful when semi-quantitative indices are determined [7].

Atherosclerosis risk factors such as hypertension, obesity, diabetes, dyslipidemia, and insulin resistance frequently accompany NAFLD [8]. Moreover, NAFLD has been recently included among the early precursors to metabolic syndrome, a clinical condition associated with a high risk of coronary heart disease [9,10,11]. However, few clinical studies have examined the association between NAFLD and coronary atherosclerosis in patients with low cardiovascular risk factors. Theoretically, the increased cardiovascular risk in patients with NAFLD might reflect the coexistence of underlying metabolic syndrome risk factors. Alternatively, NAFLD itself might confer a cardiovascular risk above and beyond that associated with the individual components of the metabolic syndrome [12].

Coronary artery disease (CAD) has been found to associate with plaque rupture, independent of the degree of coronary luminal narrowing [13,14,15]. Plaque that is vulnerable to rupture shows positive remodelling of the vessel, *i.e.*, a lipid core (soft plaque), spot calcification (calcified plaque), and a thin fibrous cap. Recent observations by intravascular US suggest that the majority of patients with ischemic heart disease have previously ruptured coronary artery plaque in non-culprit coronary arteries, in addition to infarcted-related culprit arteries [16,17,18]. Multislice coronary computed tomography angiography (cardiac CT) has been proposed as a non-invasive modality to detect coronary plaques and classify CAD [19].

Carotid atherosclerosis has been detected in patients with non-alcoholic fatty liver disease in cross-sectional studies; and more recently, carotid intima-media thickness (IMT) has been related to the severity of liver damage [20]. Retinal arteriolar abnormalities have been recognized as associating with hypertension and diabetes mellitus. Although many studies have demonstrated associations between hypertension and retinal arteriolar changes in people without diabetes [21], few have examined the associations of these arteriolar changes with other risk factors like fatty liver. The relationship of these arteriolar changes with atherosclerotic, inflammatory, and other pathogenic processes is largely unknown. More recently, Miller *et al.* showed that smaller arterial caliber may indicate an increased risk of CAD in patients with diabetes [22].

Accordingly, the purpose of the present study was to explore an association between retinal arteriolar changes and atherosclerosis (coronary plaques, carotid plaques, and IMT) in individuals with fatty liver but without hypertension and without diabetes.

## 2. Materials and Methods

This is an observational study. During the period April 2006 to June 2008, 150 individuals were referred to the cardiology unit at our medical center for cardiac CT for various clinical reasons consistent with routine care.

Inclusion criteria for participation in the study were low risk for coronary artery disease (CAD), the presence of fatty liver (liver minus spleen density ≥ −10 HU by CT), and the absence of diabetes and hypertension. Of 150 patients referred, 99 were excluded due to high risk for CAD, presence of diabetes, and/or hypertension. Of the remaining 51 patients, 29 had fatty liver disease and 22 did not. Exclusion criteria comprised severe obesity (BMI > 35; recent history of acute illness; clinical history of ischemic heart disease and cerebrovascular disease, typical chest pain, previous CAD, conventional coronary angiography, percutaneous intervention, coronary bypass grafting, renal failure, cancer; and use of drugs that may induce hepatic steatosis (such as corticosteroids, estrogens, methotrexate, amiodarone). Specific exclusion criteria for cardiac CT were high risk for CAD, the presence of multiple ectopic beats, atrial fibrillation, heart rate more than 75/min despite therapy, severe lung disease, and a history of allergic reaction to iodine-containing contrast agents.

The study was approved by the local ethics committee at Ziv Medical Center, Israel. Informed consent was obtained from each individual who met inclusion/exclusion criteria. All subjects underwent a complete family history, physical examination, and non-contrast CT of the liver with measurement of liver and spleen density. All were evaluated for markers of insulin resistance (fasting glucose and homeostasis model assessment of insulin resistance—HOMA-IR). HOMA-IR was derived from the following equation: IR = (fasting plasma glucose level mg% × 0.055) × (fasting plasma insulin level mU/L/22.5). Body mass index (BMI) was calculated as weight in kilograms divided by the square of height in meters. Obesity was determined as BMI exceeding 30 kg/m² and overweight as BMI 25–28 kg/m^2^; new diabetes onset was determined by fasting plasma glucose levels >126 mg/dL. Markers of lipotoxicity including triglyceride and cholesterol levels were obtained, as well as markers of inflammation including C-reactive protein (CRP) and fibrinogen. CRP was determined by the nephlometric method, and fibrinogen by the coagulative method of von Clauss [23,24]. Markers of oxidant-anti-oxidant status that were assessed included paraoxonase, alpha-tocopherol, and malondialdehyde (MDA). Paraoxonase activity was measured as previously described, using phenyl acetate as substrate [25]; α-tocopherol was estimated spectrophotometrically [26]. Lipid peroxidation (MDA concentration) was estimated spectrophotometrically using thiobarbituric acid assay [27].

**Figure 1:**
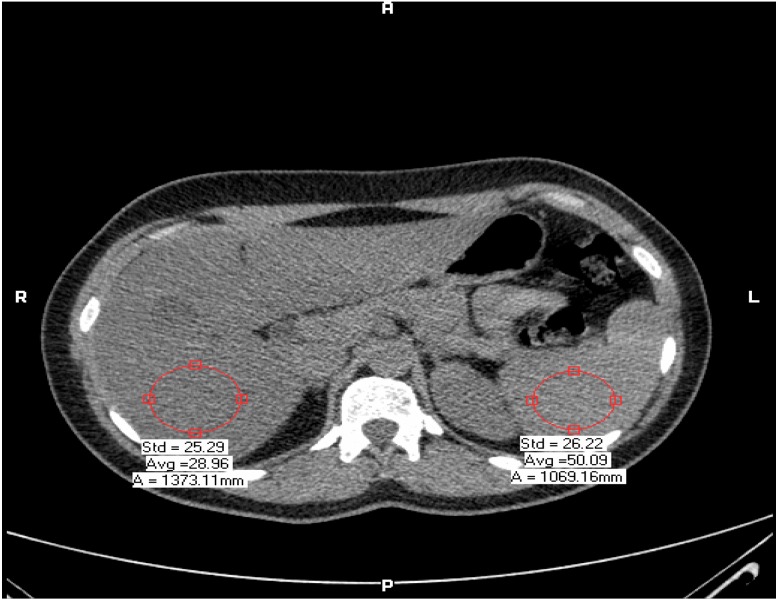
Example of fatty liver diagnosed by CT: liver minus spleen density > −10 Hounsfield units (HU).

**Figure 2:**
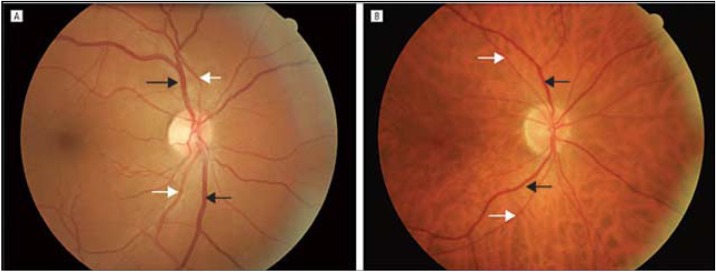
Digitized retinal photographs showing examples of low and high arteriole-to-venule ratio (AVR). (**A**), AVR = 0.789; (**B**), AVR = 0.973. Arterioles are lighter in intensity compared with venules. (Two different patients).

Hepatic steatosis was defined as liver minus spleen density > −10 Hounsfield units by CT [7,28] (Figure 1). All CT examinations were performed by the same experienced radiologist (LA, 20 years experience in radiology) blinded to the clinical status of the patients. The retinal photography procedure followed standardized methods. Briefly, after 5 min of dark adaptation, a 45°, retinalphotograph was taken of one randomly selected eye using an auto focus camera. The photograph was centered on the region of the optic disc and the macula. The photographs were digitized by a high-resolution scanner and the diameters of individual arterioles and venules coursing through a zone located one half to 1 disc diameter from the optic disc margin were measured on the computer bytrained graders who were masked to subject identity. These measurements were summarized as a retinal arteriole-to-venule ratio (AVR). The AVR accounts for magnification differences between photographs; it is characterized by normal distribution in the general population. A smaller AVR indicates narrower arterioles, since venular diameters vary little with blood pressure [20]. Intragraded and intergraded reliability coefficients for repeated AVR measurements were 0.84 and 0.79, respectively. Examples of low and high AVR are shown in Figure 2.

**Figure 3:**
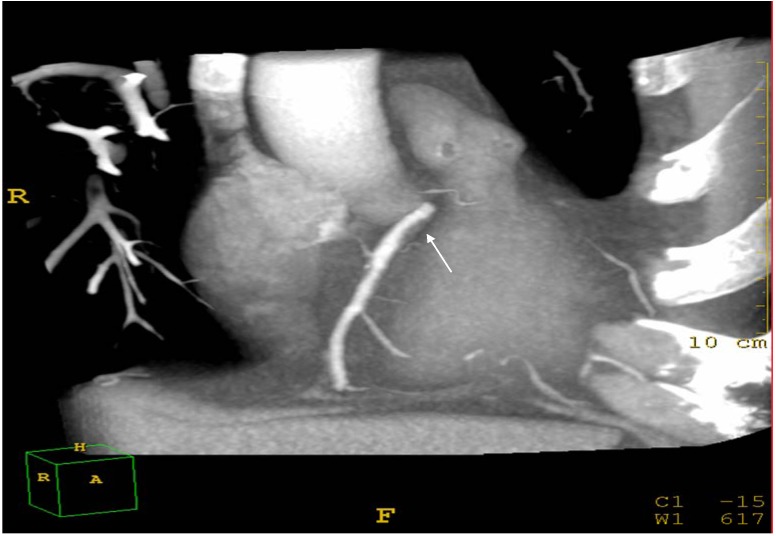
Cardiac CT: Example of soft (non calcified) coronary plaques at the proximal area of the right coronary artery (arrow).

Assessment of coronary atherosclerosis was performed according to cardiac CT protocols. The patients were studied in the supine position. A bolus of 70–90 mL iomeprol contrast medium (400 mg/mL, Iomeron 400, Bracco, Milan, Italy) was injected intravenously (4 mL/s) followed by 50 mL bolus of saline via an 18 gauge catheter placed in the antecubital vein. Scan delay was determined employing an automatic bolus test in which the region of interest was located on the ascending aorta. Patients were instructed to maintain an inspiratory breath hold while CT data and electrocardiograph (ECG) trace were acquired. A 64-row scanner with a slice thickness of 0.625 mm (Brilliance Philips Medical Systems, Andover, MA, USA) was employed. For multidetector CT angiography, retrospective ECG gating was used, with heart rate adjusted gantry rotation of 350–500 ms to enable multi-segmented reconstruction. Temporal resolution was as low as 53 ms using 0.4 rotation and adaptive multicycle reconstruction. Pitch and tube currents of 200–500 mA were determined by patients’ weight. Beta blockers (propranolol 20 mg tab) were administered orally if resting heart rate was above 70 beats per minute. If heart rate was more than 80 beats per minute the patient was excluded from the study. Raw image data sets from all acquisitions were analysed by two independent specialists (AM > 20 years experience in cardiology and LA > 20 years experience in radiology) unaware of the hepatic and lipid profile of the patients. Intra-observer and inter-observer variability was measured by two readings of each observer. The two independent observers visually graded the degree of coronary stenosis (Figure 3).

The degree of stenosis was considered significant if >50% occlusion of the arterial lumen was present [29]. Plaques were classified as soft (none calcified) and calcified on a segmental basis according to plaque features, including volume, attenuation, and calcification pattern. In case of discrepancy between readers, a consensus reading was performed. Multi-segment reconstruction was performed with 0.6 slice thickness; 0.3 mm overlap; multiple phases of 70, 75, and 80%; and ECG editing (curved multiplanar reformat).

**Figure 4:**
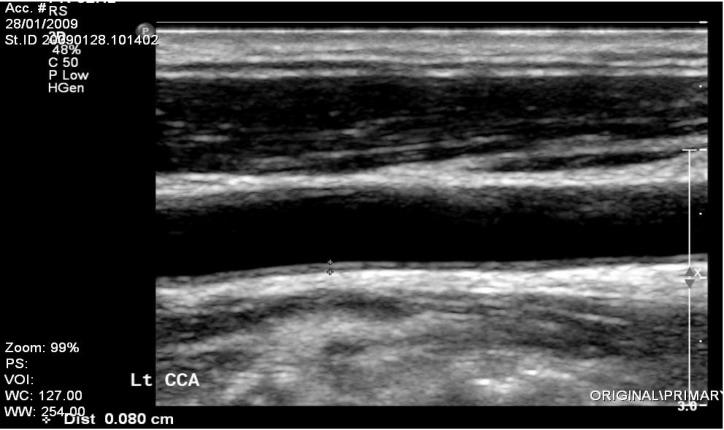
Example of abnormal intima media thickening (IMT). Normal values of IMT: (age > 50 years; women < 0.7 mm, men < 0.8 mm).

For bilateral assessment of carotid atherosclerosis, all subjects underwent carotid ultrasound for evaluation of intima-media thickness (IMT) and plaques. Carotid scans were acquired using high-resolution ultrasound (Sonos 5500, Agilent Technologies, Palo Alto, CA, USA) and a 10-MHz linear array transducer. Longitudinal views of the left and right common carotid, carotid bifurcations, and internal and external carotid arteries were obtained and recorded on super VHS tapes. All ultrasound studies (carotid and brachial flow-mediated dilation (FMD)) were analyzed off-line by specially trained technicians blinded to other study variables using the prosound system; IMT was measured over 10 mm in the far wall of the common carotid within 2 cm proximal to the bulb. The region with the thickest IMT without focal lesions was measured. Left and right carotid IMTs were averaged. Intima media area in mm^2^ was defined as: (IMT mm) × (length over which IMT was measured (20 mm)). Plaques were quantified in all carotid segments (common, internal, and external carotid arteries, Figure 4). Plaque was defined as any focal protrusion above the intima [30].

## 3. Statistical Analysis

Continuous variables are displayed as mean and standard deviation, and categorical variables as percentage. Differences in demographic and clinical variables between groups were compared using Χ^2^ analysis for categorical variables and student T-test for continuous variables. We used multiple logistic regression analysis after adjustment for possible confounding factors to assess the association among liver steatosis, markers of insulin resistance, lipotoxicity, inflammation, oxidant and anti-oxidant status, and coronary atherosclerosis (plaque and stenosis). Variables included as covariate were age, gender, LDL-cholesterol, metabolic syndrome (ATP III Guidelines) [11], and biomarkers. Results were expressed as odds ratios. Intra-observer and inter-observer agreement between readers and kappa statistics were calculated. Winstat statistic for window, version 3.0, Kalmia Co. Inc. (Cambridge, MA, USA) was used for statistical analysis.

## 4. Results

Twenty-nine subjects were diagnosed with NAFLD (without hypertension and without diabetes ) defined by CT (liver minus spleen density ≥ −10 HU), absence of alcohol use (<20 g/day), negative serology for hepatitis B or C diagnosis, negative auto-antibodies, and absence of history of another known liver disease. The control group consisted of 22 gender and age-matched individuals without NAFLD. Intra-observer and inter-observer variability for coronary artery disease (CAD) (stenosis > 50%) was 1% and 5% respectively. Baseline clinical and biochemical characteristics of NAFLD subjects and controls are shown in Table 1. Liver-spleen density was significantly lower in patients with NAFLD than in controls (−15.3 ± 8.9 *vs*. +6.8 ± 6.7, *p* < 0.001). The prevalence of smoking was similar among NAFLD patients and controls. Subjects diagnosed with NAFLD had higher triglyceride levels, HOMA values, and waist circumference measurements than controls. All 51 subjects, NAFLD patients and controls, were overweight (BMI 25–30 kg/m^2^) without statistically significant differences in BMI between the groups (Table 1). Compared with controls, NAFLD patients had a higher percentage of atherosclerotic plaques (70% *vs.* 30%, *p* < 0.001), as well as soft plaques (50% *vs.* 30%, *p* = 0.001), and greater prevalence of coronary artery disease (30% *vs.* 15%, *p* < 0.008), higher right mean IMT, lower retinal artery diameter, and lower AVR (Table 2). After adjustment for possible confounding factors that were identified in univariate analysis, multiple logistic regression analysis demonstrated that NAFLD, IMT, and retinal AVR were strongly associated with CAD, whereas CRP, a biomarker of inflammation was not (Table 3). The association of NAFLD with more severe coronary atherosclerosis remained significant also after adjustment for metabolic syndrome, age, and LDL cholesterol.

**Table 1:** Clinical and laboratory characteristic of patients with NAFLD (without hypertension and without diabetes) and controls.

	With NAFLD N = 29	Controls N = 22	*p* value NAFLD *vs.* Controls
Age (years)	53 ± 7	51 ± 10	0.3
Sex (male), n, %	86	85	0.08
BMI	30 ± 3	30 ± 4	0.08
Waist circumference (cm)	107 ± 8	103 ± 11	0.03
AST (IU/L)	27 ± 8	24 ± 6	0.05
ALT (IU/L)	36 ± 18	25 ± 10	0.005
Alk-Phos (IU/L)	74 ± 20	76 ± 18	0.3
GGT (IU/L)	37 ± 16	32 ± 18	0.05
Triglycerides (mg/dL)	209 ± 88	148 ± 70	0.005
LDL cholesterol mg/dL)	117 ± 36	113 ± 33	0.3
HDL cholesterol (mg/dL)	43 ± 12	46 ± 12	0.2
Metabolic syndrome (%)	62	31	0.008
Liver-Spleen Density (HU)	−15.3 ± 8.9	+6.8 ± 6.7	0.001

**Table 2:** Cardiac CT, carotis Doppler ultrasound, and retinal angiography findings and biomarkers in patients with NAFLD and controls.

	with NAFLD N = 29	Controls N = 22	*p* value NAFLD *vs.* Controls
Coronary artery disease (stenosis > 50%)	30%	15%	0.001
Coronary Plaques%Soft Plaques	70%50%	30%30%	0.010.01
IMT, mean Right Carotis (mm)	0.90 ± 0.19	0.8 ± 0.10	0.03
Left Carotis (mm)	0.98 ± 0.30	0.8 ± 0.10	0.27
Mean Carotis Plaques%	60%	40%	0.01
Retinal artery diameter	92.4 ± 13.08	100.56 ± 7.1	0.04
Retinal vein diameter	136.37 ± 14.42	141.5 ± 7.05	0.2
AVR	0.66 ± 0.06	0.71 ± 0.02	0.01
CRP	0.41 ± 0.9	0.30 ± 0.3	0.02
MDA(mM)	0.09 ± 0.05	0.11 ± 0.05	0.09
Paroxonase (mM/min)	0.55 ± 0.1	0.57 ± 0.1	0.4
HOMA	4.0 ± 3.4	2.0 ± 1.0	0.001

Intima media thickness (IMT), arteriole-to-venule ratio (AVR); malondialdehyde (MDA), C-reactive protein (CRP), homeostasis model assessment of insulin resistance HOMA.

**Table 3:** Multivariate model for prediction of coronary artery disease in patients with fatty liver.

	OR *	±95% CI	*p*
Fatty Liver (L-S density < −10 HU)	2.5	1–4.0	0.001
IMT (>0.80 mm)	2.3	0.9–3	0.001
Retinal AVR (>0.7)	1.5	0.5–1.5	0.01
HOMA (>3.0)	0.8	0.5–1.5	0.08
CRP (>0.5 mg/dL)	0.7	0.5–0.9	0.4
Metabolic syndrome (present/absent)	1.2	0.9–3.5	0.03

* Odds ratios (OR) are adjusted for gender, age, smoking habits, metabolic syndrome, diabetes, BMI, and levels of ALT, HDL and LDL-cholesterol, triglycerides, and fasting glucose.

## 5. Discussion

This study demonstrated that subjects with NAFLD had significant retinal vascular changes (lower AVR), and a higher prevalence of cardiovascular diseases than controls. Moreover, intima media thickness and retinal AVR were found to be strongly associated with CAD in patients with NAFLD, independent of metabolic syndrome. Retinal arterial caliber has previously been shown to be associated with blood pressure; retinal venular caliber has been associated with smoking, atherosclerosis, lipid levels, obesity, and inflammation [31]. Importantly, our patients were non-diabetic and non-hypertensive. 

The independent association between AVR and coronary plaques reflects microvascular rather than macrovascular processes. Our findings are supported by cross-sectional data from the United States National Health Examination Survey. An explanation for lower AVR as an indicator of more severe coronary artery disease remains to be determined. One possibility is that patients with NAFLD have increased endothelial dysfunction (IMT > 0.7 mm, retinal AVR < 0.7), which results in increased incidence of atherosclerosis, and ultimately in significant coronary artery disease [32].

Retinal vascular caliber changes reflect cumulative responses to aging, cardiovascular risk factors, inflammation, nitric oxide-dependent endothelial dysfunction, and other processes [32]. Recent epidemiological studies have shown that changes in retinal arteriolar and venular caliber size may reflect the differential effects of a range of systemic, environmental, and genetic risk factors [33]. Narrower retinal arteriolar caliber and smaller arteriovenous ratio are associated with older age and higher levels of blood pressure and obesity; and predict the incidence of diabetes and coronary heart disease. In contrast, wider retinal venular caliber is associated with younger age; impaired fasting glucose and diabetes; dyslipidemia; obesity; and systemic markers of inflammation, endothelial dysfunction, and cigarette smoking; and predicts the risk of stroke and coronary heart disease [32]. Whether fatty liver *per se* increases the risk of cardiovascular disease or reflects liver systemic insulin resistance is unknown. Studies suggest that both mechanisms are involved [34,35]. NAFLD is a strong risk factor for endothelial dysfunction [36]. While oxidative stress may influence the risk of cardiovascular disease, we did not observe significant differences in oxidation biomarkers between patients with NAFLD and controls.

Systemic inflammation may account for increased coronary plaques. Our study showed a significant difference in CRP levels between patients with severe fatty liver and controls. Recently, it has been shown that patients with elevated CRP and receiving rosovastatin had 48% reduction in stroke prevalence, 46% reduction in the need for intervention to reopen blocked blood vessels, and a 20% reduction in all-cause mortality (JUPITER Trial) [37]. Increases in CRP or TNF-alpha are key to the control of metabolism and inflammation, and an independent determinant of NAFLD [37]. Other potential mechanisms by which fatty liver may increase cardiovascular risk beyond that imposed by the metabolic syndrome are lipotoxicity and increased circulating IGF-1 and IGFBP-3 [38]. Lipotoxicity may account for the increased risk of coronary plaque by a rise in serum oxidized fatty acid, which has been associated with increased IMT [39]. Unfortunately, we did not measure the levels of free fatty acids and oxidized LDL in our study population.

This case controlled study suggests that fatty liver, IMT, and retinal AVR are the strongest predictors of coronary plaques independent of the metabolic syndrome. The clinical implication of this finding is that patients with NAFLD should be screened regularly for other cardiovascular risk factors and that the presence of fatty liver may improve stratification of these patients. This notion is reinforced by the fact that NAFLD may occur in children, preceding the onset of cardiovascular risk factors [40]. Importantly, our findings were independent of BMI, even though obesity and metabolic syndrome have been shown to be the main risk factors for fatty liver [41]. The majority of patients in the current study had vulnerable soft plaques. Recently, Pundziute *et al.* reported the prognostic value of cardiac CT in known and suspected coronary artery diseases [42]. They demonstrated that non obstructive lesions were an independent predictor of future cardiac events. Considering the fact that coronary occlusion and myocardial ischemia may frequently arise from mild to moderate coronary stenosis [43,44], identification of coronary artery plaques in NAFLD patients seems valuable for risk stratification and for guiding therapy. The detection of coronary plaques in patients with NAFLD may suggest the need for pharmacological therapy with insulin sensitizing agents, statins, or RAS inhibitors [45,46].

The significant association we observed between IMT and HOMA-IR (*r* = 0.8; *p* < 0.001) in the overall series and in controls is consistent with the association that has been demonstrated between increased fasting glucose and impaired glucose tolerance) and increased carotid IMT [47]. Interestingly, a moderately increased risk for cardiovascular events in patients with type 2 diabetes and associated non-alcoholic fatty liver disease compared with those without non-alcoholic fatty liver disease was reported in a large prospective study [47]. The same authors found IMT and alanine-aminotransferase to be higher in patients with non-alcoholic fatty liver disease than in controls, with the highest values in non-alcoholic steatohepatitis [35,48]. The pathogenesis of retinal arteriolar changes (focal narrowing, AVR, and arteriovenous nicking) in patients with NAFLD is unknown. These three changes could be related to hypertension, but none of our patients had hypertension. Inflammatory factors may contribute to narrowing, and vascular endothelial dysfunction may contribute to nicking [49]. Atherosclerosis, a disease of large arteries, is a major cause of CAD and stroke. Arterial narrowing, which is now shown to occur independent of atherosclerosis, might predict ischemic heart and cerebral vascular diseases [49]. Retinal arterial narrowing has been considered an early sign of hypertension [50]. In our study, lower AVR was strongly associated with coronary artery disease in patients with NAFLD. This suggests a diffuse effect of disease in the coronary and in the carotid artery rather than a local process. Retinal artery narrowing shares many risk factors with atherosclerosis. These include hypertension, smoking, and acute inflammation [51,52]. Inflammation may explain the association between low AVR and fatty liver, since cytokines causing the innate inflammatory response may originate in adipose tissues. Understanding the pathogenesis of a low AVR is important because it may explain in part, the pathogenesis of coronary or carotid plaques. The impaired microvascular function is thought to be a key factor in the development of atherosclerosis. From practical point of view, smaller retinal AVR have an added value compared to others methods for the determination of cardiovascular risk; it is simple, cost effective, accurate, and help in the decision making about risk of atherosclerosis that other methods do not cover [53].

Limitations of our study include the small sample size which prevent detailed analysis of the role of age, gender and gallstones in mediating the link between NAFLD and vascular disease [54], the absence of liver biopsies as the gold standard for the diagnosis of fatty liver and NASH and whether its it is pure steatosis rather than NASH that increase cardiovascular risk [55], and limited imaging quality which does not appear useful in understanding the mechanism involved in the pathogenesis of vascular damage in all thosde individuals with NAFLD and coronary atherosclerosis [56]. An additional limitation was that participants who underwent laser photocoagulation (diabetic and hypertensive) had to be excluded from the analysis, as the procedure is known to decrease vessel diameter. These persons were likely the patients with the most advanced micro vascular disease, so their exclusion may have introduced a selection bias in which the patients at highest risk for CAD were not addressed. However, it is likely that excluding these patients actually attenuated the relationship between retinal arteriolar and venular caliber and CAD, rather than artificially leading to the results observed here. Further studies incorporating noninvasive stress test are needed. We do not know whether the data in individuals with fatty liver with hypertension and diabetes are different from the present data

In conclusion, narrow retinal artery caliber and small retinal AVR may indicate increased risk for CAD and carotid atherosclerosis in patients with NAFLD even without hypertension or type 2 diabetes. Additional studies are needed to examine the role of retinal microvascular changes in the pathogenesis of CAD in patients with NAFLD.

## 6. Conclusions

The conclusions of this work may be summarized as follows:

### Advances in Knowledge

1. Carotid atherosclerosis has been documented in patients with non-alcoholic fatty liver disease; and more recently, carotid intima-media thickness has been related to the severity of liver damage.
2. Retinal arteriolar abnormalities have been recognized as associating with hypertension and diabetes mellitus.

### Implications

1. Smaller retinal arterial caliber may indicate an increased risk of cardiovascular disease in patients with NAFLD and may allow intervention at earlier stage.
2. More individuals from the general population with sub clinical coronary artery diseases will be detected at earlier stage when fatty liver and retinal vascular changes are identified.

### Summary

Narrower retinal artery caliber and smaller retinal AVR may indicate increased risk for cardiovascular disease in patients with NAFLD, even without hypertension or diabetes.

## References

[1] Angulo P. (2002). Nonalcoholic fatty liver disease. N. Engl. J. Med..

[2] Williams C.D., Stengel J., Asike M.I., Torres D.M., Shaw J., Contreras M., Landt C.L., Harrison S.A. (2011). Prevalence of nonalcoholic fatty liver disease and nonalcoholic steatohepatitis among a largely middle-aged population utilizing ultrasound and liver biopsy: A prospective study. Gastroenterology.

[3] McCullough A.J. (2004). The clinical features, diagnosis and natural history of nonalcoholic fatty liver disease. Clin. Liver Dis..

[4] Perseghin G., Lattuada G., de Cobelli F., Ntali G., Esposito A., Burska A., Belloni E., Canu T., Ragogna F., Scifo P. (2006). Serum resistin and hepatic fat content in nondiabetic individuals. J. Clin. Endocrinol. Metab..

[5] Assy N., Bekirov I., Mejritsky Y., Solomon L., Szvalb S., Hussein O. (2005). Association between thrombotic risk factors and extent of fibrosis in patients with non-alcoholic fatty liver diseases. World J. Gastroenterol..

[6] Ruhl C.E., Everhart J.E. (2003). Determinants of the association of overweight with elevated serum alanine aminotransferase activity in the United States. Gastroenterology.

[7] Ballestri S., Lonardo A., Romagnoli D., Carulli L., Losi L., Day C.P., Loria P. (2012). Ultrasonographic fatty liver indicator, a novel score which rules out NASH and is correlated with metabolic parameters in NAFLD. Liver Int..

[8] Akahoshi M., Amasaki Y., Soda M., Tominaga T., Ichimaru S., Nakashima E., Seto S., Yano K. (2001). Correlation between fatty liver and coronary risk factors: A population study of elderly men and women in Nagasaki, Japan. Hypertens. Res..

[9] Anstee Q.M., Targher G., Day C.P. (2013). Progression of NAFLD to diabetes mellitus, cardiovascular disease or cirrhosis. Nat. Rev. Gastroenterol. Hepatol..

[10] Malik S., Wong N.D., Franklin S.S., Kamath T.V., L’Italien G.J., Pio J.R., Williams G.R. (2004). Impact of the metabolic syndrome on mortality from coronary heart disease, cardiovascular disease, and all causes in United States adults. Circulation.

[11] Expert Panel on Detection, Evaluation, and Treatment of High Blood Cholesterol in Adults (2001). Executive summary of the third report of the National Cholesterol Education Program (NCEP) Expert Panel on Detection, Evaluation, and Treatment of High Blood Cholesterol in Adults (Adult Treatment Panel III). JAMA.

[12] Hamaguchi M., Kojima T., Takeda N., Nagata C., Takeda J., Sarui H., Kawahito Y., Yoshida N., Suetsugu A., Kato T. (2007). Nonalcoholic fatty liver disease is a novel predictor of cardiovascular disease. World J. Gastroenterol..

[13] Davis M., Thomas A. (1984). Thrombosis and acute coronary artery lesions in sudden cardiac ischemic death. N. Engl. J. Med..

[14] Fuster V., Badimon L., Badimon J.J., Chaesebro J.H. (1992). The pathogenesis of coronary artery disease and the acute coronary syndrome. N. Engl. J. Med..

[15] Theroux P., Fuster V. (1998). Acute coronary syndrome: Unstable angina and non-Q-wave myocardial infarction. Circulation.

[16] Riouful G., Finet G., Ginon I., André-Fouët X., Rossi R., Vialle E., Desjoyaux E., Convert G., Huret J.F., Tabib A. (2002). Multiple atherosclerotic plaques rupture in acute coronary syndrome. Circulation.

[17] Hong M.K., Mintz G.S., Lee C.W., Kim Y.H., Lee S.W., Song J.M., Han K.H., Kang D.H., Song J.K., Kim J.J. (2004). Comparison of coronary plaque rupture between stable angina and acute myocardial infarction: A three-vessel intravascular US study in 235 patients. Circulation.

[18] Tanaka A., Shimada K., Sano T., Namba M., Sakamoto T., Nishida Y., Kawarabayashi T., Fukuda D., Yoshikawa J. (2005). Multiple plaque rupture and C-reactive protein in acute myocardial infarction. J. Am. Coll. Cardiol..

[19] Akabame S., Hamaguchi M., Tomiyasu K., Tanaka M., Kobayashi-Takenaka Y., Nakano K., Oda Y., Yoshikawa T. (2008). Evaluation of vulnerable coronary plaques and non-alcoholic fatty liver disease (NAFLD) by 64-detector multislice computed tomography (MSCT). Circ. J..

[20] Targher G., Bertolini L., Padovani R., Rodella S., Zoppini G., Zenari L., Cigolini M., Falezza G., Arcaro G. (2006). Relations between carotid artery wall thickness and liver histology in subjects with nonalcoholic fatty liver disease. Diabetes Care.

[21] Hubbard L.D., Brothers R.J., King W.N., Clegg L.X., Klein R., Cooper L.S., Sharrett A.R., Davis M.D., Cai J. (1999). Methods for evaluation of retinal microvascular abnormalities associated with hypertension/sclerosis in the Atherosclerosis Risk in Communities (ARIC) Study. Ophthalmology.

[22] Miller R.G., Prince C.T., Klein R., Orchard T.J. (2009). Retinal vessel diameter and the incidence of coronary artery disease in type 1 diabetes. Am. J. Ophthalmol..

[23] Montagne P., Laroche P., Cuillière M.L., Varcin P., Pau B., Duheille J. (1992). Particle-enhanced nephelometric immunoassay for human C-reactive protein. J. Clin. Lab. Anal..

[24] Clauss A. (1957). Gerinnungsphysiologische Schnellmethode zur Bestimmung des Fibrinogens. Rapid physiological coagulation method in determination of fibrinogen. Acta Haematol..

[25] Gan K.N., Smolen A., Eckerson H.W., la Du B.N. (1991). Purification of human serum paraoxonase/arylesterase. Evidence for one esterase catalyzing both activities. Drug Metab. Dispos..

[26] Baker H., Frank O., de Angelis B., Feingold S. (1980). Plasma tocopherol in man at various time intervals after ingesting free or acetylated tocopherol. Nutr. Rep. Int..

[27] Yagi K. (1987). Lipid peroxides and human diseases. Chem. Phys. Lipids.

[28] Limanond P., Raman S., Lassman C. (2004). Macrovesicular hepatic steatosis in living related liver donors: Correlation between CT and histologic findings. Radiology.

[29] Schroeder S., Kopp A.F., Baumbach A., Meisner C., Kuettner A., Georg C., Ohnesorge B., Herdeg C., Claussen C.D., Karsch K.R. (2001). Noninvasive detection and evaluation of atherosclerotic coronary plaques with multislice computed tomography. J. Am. Coll. Cardiol..

[30] De Groot E., Hovingh G.K., Wiegman A., Duriez P., Smit A.J., Fruchart J.C., Kastelein J.J. (2004). Measurement of arterial wall thickness as surrogate marker for atherosclerosis. Circulation.

[31] Ikram M.K., de Jong F.J., Vingerling J.R., Witteman J.C., Hofman A., Breteler M.M., de Jong P.T. (2004). Are retinal arteriolar or venular diameters associated with markers for cardiovascular disorders? The Rotterdam Study. Invest. Ophthalmol. Vis. Sci..

[32] Sun C., Wang J.J., Mackey D.A., Wong T.Y. (2009). Retinal vascular caliber: Systemic, environmental, and genetic associations. Surv. Ophthalmol..

[33] Gillum R.F. (1991). Retinal arteriolar findings and coronary heart disease. Am. Heart J..

[34] Samuel V.T., Liu Z.X., Qu X., Elder B.D., Bilz S., Befroy D., Romanelli A.J., Shulman G.I. (2004). Mechanism of hepatic insulin resistance in non-alcoholic fatty liver disease. J. Biol. Chem..

[35] Nseir W., Shalata A., Marmor A., Assy N. (2011). Mechanisms linking nonalcoholic fatty liver disease with coronary artery disease. Dig. Dis. Sci..

[36] Villanova N., Moscatiello S., Ramilli S., Bugianesi E., Magalotti D., Vanni E., Zoli M., Marchesini G. (2005). Endothelial dysfunction and cardiovascular risk profile in nonalcoholic fatty liver disease. Hepatology.

[37] Ridker P.M., Danielson E., Fonseca F.A., Genest J., Gotto A.M., Kastelein J.J., Koenig W., Libby P., Lorenzatti A.J., MacFadyen J.G. (2008). Rosuvastatin to prevent vascular events in men and women with elevated C-reactive protein. N. Engl. J. Med..

[38] Kawachi S., Takeda N. (2005). Circulating insulin-like growth factor-1 and insulin-like growth factor binding protein-3 are associated with early carotid atherosclerosis. Arterioscler. Thromb. Vasc. Biol..

[39] Björntorp P. (1990). Portal adipose tissue as a generator of risk factors for cardiovascular disease and diabetes. Arteriosclerosis.

[40] Schwimmer J.B., Deutsch R., Kahen T., Lavine J.E., Stanley C., Behling C. (2006). Prevalence of fatty liver in children and adolescents. Pediatrics.

[41] Licata G., Argano C., di Chiara T., Parrinello G., Scaglione R. (2006). Obesity: A main factor of metabolic syndrome?. Panminerva Med..

[42] Pundziute G., Schuijf J.D., Jukema J.W., Boersma E., de Roos A., van der Wall E.E., Bax J.J. (2007). Prognostic value of multislice computed tomography coronary angiography in patients with known or suspected coronary artery disease. J. Am. Coll. Cardiol..

[43] Girould D., Li J.M., Urban B., Rutishhauer W. (1992). Relation of the site of acute myocardial infarction to the most severe coronary arterial stenosis at prior angiography. Am. J. Cardiol..

[44] Adelman E.L., Corly S.D., Fisher L.D., Chaitman B.R., Faxon D.P., Foster E.D., Killip T., Sosa J.A., Bourassa M.G. (1993). Five-year angiographic follow up of factors associated with progression of coronary artery disease in the coronary artery surgery study (CASS). CASS participating investigators and staff. J. Am. Coll. Cardiol..

[45] Grundy S.M., Cleeman J.I., Merz C.N., Brewer H.B., Clark L.T., Hunninghake D.B., Pasternak R.C., Smith S.C., Stone N.J., National Heart, Lung, and Blood Institute (2004). Implication of recent clinical trials for the National cholesterol education program adult treatment panel III guidelines. Circulation.

[46] Smith S.C., Allen J., Blair S.N., Bonow R.O., Brass L.M., Fonarow G.C., Grundy S.M., Hiratzka L., Jones D., Krumholz H.M. (2006). AHA/ACC guidelines for secondary prevention for patients with coronary and other atherosclerotic vascular disease: 2006 Update: Endorsed by the National Heart, Lung, and Blood Institute. Circulation.

[47] Targher G., Bertolini L., Poli F., Rodella S., Scala L., Tessari R., Zenari L., Falezza G. (2005). Nonalcoholic fatty liver disease and risk of future cardiovascular events among type 2 diabetic patients. Diabetes.

[48] Fracanzani A.L., Burdick L., Raselli S., Pedotti P., Grigore L., Santorelli G., Valenti L., Maraschi A., Catapano A., Fargion S. (2008). Carotid artery intima-media thickness in nonalcoholic fatty liver disease. Am. J. Med..

[49] Klein R., Sharrett A.R., Klein B.E., Chambless L.E., Cooper L.S., Hubbard L.D., Evans G. (2000). Are retinal arteriolar abnormalities related to atherosclerosis? The atherosclerosis risk in communities study. Arterioscler. Thromb. Vasc. Biol..

[50] Mitsutake R., Niimura H., Miura S., Zhang B., Iwata A., Nishikawa H., Kawamura A., Kumagai K., Shirai K., Matsunaga A., Saku K. (2006). Clinical significance of the coronary calcification score by multidetector row computed tomography for the evaluation of coronary stenosis in Japanese patients. Circ. J..

[51] Walsh J.B. (1982). Hypertensive retinopathy. Description, classification, and prognosis. Ophthalmology.

[52] Ross R. (1999). Atherosclerosis—An inflammatory disease. N. Engl. J. Med..

[53] Tabatabaee A., Asharin M.R., Dehghan M.H., Pourbehi M.R., Nasiri-Ahmadabadi M., Assadi M. (2013). Retinal vessel abnormalities predict coronary artery diseases. Perfusion.

[54] Feitosa M.F., Reiner A.P., Wojczynski M.K., Graff M., North K.E., Carr J.J., Borecki I.B. (2013). Sex-influenced association of nonalcoholic fatty liver disease with coronary heart disease. Atherosclerosis.

[55] Lonardo A., Sookoian S., Chonchol M., Loria P., Targher G. (2013). Cardiovascular and systemic risk in nonalcoholic fatty liver disease—Atherosclerosis as a major player in the natural course of NAFLD. Curr. Pharm. Des..

[56] Ballestri S., Meschiari E., Baldelli E., Musumeci F.E., Romagnoli D., Trenti T., Zennaro R.G., Lonardo A., Loria P. (2013). Relationship of serum fetuin—A levels with coronary atherosclerotic burden and NAFLD in Patients undergoing elective coronary angiography. Metab. Syndr. Relat. Disord..

